# Mathematical Modeling Finds Disparate Interferon Production Rates Drive Strain-Specific Immunodynamics during Deadly Influenza Infection

**DOI:** 10.3390/v14050906

**Published:** 2022-04-27

**Authors:** Emily E. Ackerman, Jordan J. A. Weaver, Jason E. Shoemaker

**Affiliations:** 1 Department of Chemical & Petroleum Engineering, University of Pittsburgh, Pittsburgh, PA 15260, USA; eea16@pitt.edu (E.E.A.); jjw102@pitt.edu (J.J.A.W.); 2 McGowan Institute for Regenerative Medicine, University of Pittsburgh, Pittsburgh, PA 15260, USA; 3 Department of Computational and Systems Biology, University of Pittsburgh, Pittsburgh, PA 15260, USA

**Keywords:** viral strains, ODE modeling, systems biology

## Abstract

The timing and magnitude of the immune response (i.e., the immunodynamics) associated with the early innate immune response to viral infection display distinct trends across influenza A virus subtypes in vivo. Evidence shows that the timing of the type-I interferon response and the overall magnitude of immune cell infiltration are both correlated with more severe outcomes. However, the mechanisms driving the distinct immunodynamics between infections of different virus strains (strain-specific immunodynamics) remain unclear. Here, computational modeling and strain-specific immunologic data are used to identify the immune interactions that differ in mice infected with low-pathogenic H1N1 or high-pathogenic H5N1 influenza viruses. Computational exploration of free parameters between strains suggests that the production rate of interferon is the major driver of strain-specific immune responses observed in vivo, and points towards the relationship between the viral load and lung epithelial interferon production as the main source of variance between infection outcomes. A greater understanding of the contributors to strain-specific immunodynamics can be utilized in future efforts aimed at treatment development to improve clinical outcomes of high-pathogenic viral strains.

## 1. Introduction

Infections with different influenza A viruses reveal distinct trends in the observed timing and magnitude of immune system dynamics, which correlate to the severity of clinical outcomes [1]. Seasonal influenza viruses, usually of the H1N1 subtype, cause ~700,000 hospitalizations and ~56,000 deaths in the US annually [2]. Occasionally, high pathogenic subtypes emerge, which can result in deadly, worldwide pandemics such as the 1918 Spanish Flu and 1968 Flu pandemics. Of particular concern is the threat that avian H5N1 influenza viruses pose to public health [3]. An estimated 60% of human H5N1 infections end in death, the majority of which unexpectedly occur in those under the age of 65 [4]. Infections with H5N1 viruses are characterized by higher viral loads, longer viral clearance times, and increased levels of inflammation and tissue damage in comparison with low-pathogenic influenza viruses [5].

Although it remains unclear how H5N1 and other highly pathogenic viruses induce a more severe inflammatory response, there are several potential explanations. One possibility is that H5N1 viruses replicate more quickly, and that observed differences in the immune response are driven primarily by the viral replication rate [6]. Another possibility is that H5N1 viruses may antagonize the immune system differently during the early stages of infection. A specific candidate mechanism involves the influenza virus’ nonstructural protein 1 (NS1). NS1 is well-established as an antagonist of intracellular immune signaling through the inhibition of retinoic acid-inducible gene I (RIG-I) activity, which leads to a delayed type-I interferon response [7,8]. By introducing mutations to the NS1 protein, some studies have shown that the NS1 protein of H5N1 viruses may more strongly antagonize cellular antiviral responses [9,10]. Another factor that may contribute to the H5N1 virus’ enhanced pathogenicity is that H5N1 can more readily infect lung resident macrophages, though there is conflicting evidence on whether infected macrophages lead to enhanced inflammation [11] or not [12]. Given the many factors contributing to H5N1′s pathogenicity, there is an opportunity to use dynamical mathematical modeling to analyze time-course infection data and identify the processes (factors) that differ between infections with different viruses.

Dynamic mathematical models have been used to better understand the mechanisms driving in vitro and in vivo immunodynamics observed during influenza infection (see [13,14] for reviews of some relevant models). To date, most mathematical models of influenza infection consist of ordinary differential equations (ODEs) that systematically link virus replication and the availability of host target cells (cells that can be infected) to intracellular immune signaling (interferon responses) and/or immune cell activity. These models have been used to explore a variety of areas: to provide possible explanations as to why a double peak in the viral load may be observed [15], to prioritize therapeutic targets to optimally reduce inflammation while controlling viral load [15,16,17,18], to provide evidence that interferon paracrine signaling is the primary factor regulating hypercytokinemia [19], and to determine why viral titers rebound during bacterial co-infection [20]. Separately, agent-based models (ABMs), a rule-based approach that treats each cell as an individual entity while considering spatial effects and stochasticity, have been used to reveal the optimal experimental conditions for examining infection-induced interferon production, to quantify the benefits of noisy intracellular immune signaling [21], and to elucidate the effect of spatial aspects on infection outcomes [22]. An engineering-based approach that employed a reduced ODE model of virus replication, and treated measurements of key immune factors as system inputs, suggested that increased levels of interferon-α/β promoted slower viral growth, and limited immune cell stimulation in aged mice [23]. As in these previous studies, mathematical modeling is a knowledge-driven, integrative approach well-suited to explore the regulatory mechanisms responsible for the differences observed between mild and severe influenza infections.

To elucidate the biological mechanisms that contribute to the distinct immunodynamics observed between H1N1 and H5N1 influenza virus infections, we developed mechanism-based, dynamic mathematical models of the innate immune response, and performed several parameterizations to identify the biological processes (parameters) that are most likely to be differentially regulated between the two infections. The model parameters were fit to viral load and immunologic data from mice that had been infected with either an H1N1 or H5N1 virus. Comparing model fits to the data using the Akaike Information Criteria (AIC) suggests that the optimal model is achieved when the parameter representative of the production rate of interferon is distinct between the two infections. Using parameterization to test the contribution of macrophage activity in interferon production and viral suppression reveals that the inclusion of these mechanisms negatively impacts model quality. In total, this modeling-based approach determines that the distinct rate of interferon induction in H5N1 infections is the most likely candidate mechanism for explaining the distinctive immune response observed in H5N1 infections.

## 2. Materials and Methods

### 2.1. Model Development Rationale and Equations

Studies have established that many innate immune processes are differentially regulated in mild and severe influenza infections [24,25,26,27,28,29]. As such, we focused on developing simple models of the early immune response. We first describe the relevant immunology, and then describe how virus replication and innate immunity are mathematically modeled.

Lung epithelial cells, as well as lung-resident innate immune cells (i.e., macrophages and neutrophils), display pattern recognition receptors to detect viral RNA at the site of infection [30,31]. Pattern-recognition toll-like receptors 7 and 9 (TLR7/9) [32], retinoic acid-inducible gene I (RIG-I) [33], and the pro-inflammatory NF-kB [34] pathway work in concert to activate the type-I interferon response [8]. These pattern recognition receptors are antagonized by the invading virus to strain-specific degrees [35,36]. Interferons induce the transcription of interferon-stimulated genes (ISGs) that are responsible for establishing an antiviral state in the cells near infected cells [37], and activating several components of the immune system. Studies suggest that the timing of the type-I interferon response is key in limiting viral replication and recruiting an appropriate pro-inflammatory response [38,39,40]. Induction of interferon production is also partially responsible for regulating the activity of innate immune cells such as macrophages and neutrophils. The precise role of these immune cells in viral clearance is still debated: though macrophages can engage in several inflammatory processes [41] and are important for enhancing interferon induction [42], they may also be targeted for infection by highly pathogenic viruses such as H5N1 influenza, altering their overall activity [11,12].

A three-state ODE model, referred to as Model 1, was developed using the immunological knowledge of the early innate immune response to a primary influenza A virus infection (i.e., the animal’s first exposure, no antibodies for the virus present) described above (See Section 3 for a schematic of the model contained in Equations (1)–(3)). The units of each state (*V*, *I*, and *M*) are discussed in Section 2.2.

(1) $$\frac{dV}{dt}=r_{V,V}V\left(1-\frac{V}{K_{V,V}}\right)-r_{V,I}V\;I-r_{V,M}V\;M-d_{V}V$$

(2) $$\frac{dI}{dt}=r_{I,V}V+r_{I,M}M-d_{I}I$$

(3) $$\frac{dM}{dt}=\frac{r_{\;M,I\;}I^{n}}{K_{\;M,I}+I^{n}}-d_{M}M$$

Virus production is modeled in Equation (1). *V*, the concentration of virus in lung tissue, is modeled as logistic growth with a constant of proportionality, *r_V,V_*, and a carrying capacity, *K_V,V_*. This form of virus production was selected over target-cell-based modeling approaches because data concerning the number of available target cells in the lung are not available, limiting the viability and accuracy of training a model. The effect of interferon-regulated inhibition of virus replication is modeled using mass action kinetics, where *r_V,I_* is the corresponding rate constant. The inhibition of virus production via macrophage is also modeled with mass-action kinetics, where *r_V,M_* is the rate constant. Virus degrades at a rate, *d_V_*.

Type-I interferon production is modeled by Equation (2), where *I* is the concentration of interferon in the lung. Interferon is produced at a rate, *r_I,V_*, relative to viral load, and decays at rate, *d_I_*. Upregulation of interferon production via macrophages was modeled as a first-order mass-action kinetic with a rate, *r_I,M_*.

Macrophage production is modeled in Equation (3), where *M* is the number of macrophages in the lung. Interferon induction of macrophage production is modeled using a Hill kinetic with a production rate, *r_M,I_*, and an apparent dissociation constant, *K_M,I_*. Instead of the classic interpretation of the Hill coefficient, *n*, as cooperativity in ligand binding [43], it can be interpreted in this context as an activation threshold representing the threshold of interferon needed to induce macrophage production. This is similar to the activation threshold that must be exceeded to induce T cell cytokine production [44,45]. The parameter, *K_M,I_*, is not raised to the Hill-like coefficient, *n*, to improve parameter fitting. Macrophage decays at a rate of *d_M_*.

Equations (1)–(3) define the dynamic behavior of Model 1. We also developed reduced models, Models 2–4, in which select interactions were removed to consider additional hypotheses on how the immune system in the lung might be regulated. For example, in CCR2-/- mice, there is conflicting evidence concerning whether inhibited macrophage infiltration into the lung of infected mice affects viral load [46,47]. In addition, although macrophage upregulation of interferon is well justified, it is not guaranteed that parameters associated with this interaction can be estimated from the data. In Model 2, *M* induction of *I* is removed. In Model 3, *M* inhibition of *V* is removed. In Model 4, both *M* induction of *I* and *M* inhibition of *V* are removed. These models were each fit to the experimental data to determine which model (and, therefore, which combination of biological processes) optimally fits the data based on the goodness of fit and the number of parameters estimated (degrees of freedom, DoF).

### 2.2. Experimental Data Collected from Literature and Relating the Data to the Model

Measurements of the viral load, interferon concentration, and a surrogate measurement of macrophage counts were collected and organized from Shoemaker et al. [1]. Briefly, female C57BL/6J mice were infected with a low pathogenic A/Kawasaki/UTK-4/09 H1N1 virus (H1N1) or high pathogenic A/Vietnam/1203/04 H5N1 virus (H5N1) at 10^5^ PFU. A control group was mock-infected with PBS. At 14 time points spanning the first week of infection, three animals per infection group were sacrificed. Their lungs were harvested and analyzed by a variety of techniques to quantify the viral load and the state of the immune system. The H5N1-infected animals died between days 5 and 7. As such, only the first 13 measurements spanning days 0–5 are included in this work. In all, 234 measurements (78 for each model state) were collected and organized for model parameterization.

The specific measurements used from [1] and their relationship to the mathematical models are as follows: Viral titers were determined via plaque assay, resulting in units of plaque-forming units per mg of lung tissue (PFU/mg). In Equation (1), *V* is the log_10_ of PFU/mg. To represent the change in interferon concentration over time in Equation (2), log_2_ fold change of the gene expression of *Ifnb1* relative to mock-infected, time-matched samples (unitless) was used. Full details on normalizing the gene expression can be found in the original work [1]. Whole lung macrophage counts were determined at only four time points in the original work, spread across several days [1]. As a result, the concentration of *MCP*1 (measured using ELISA assay) was selected to act as a surrogate measurement of macrophage cell count (*M*). Supplementary Figure S1 shows a linear regression of the log_10_ macrophage cell count and log_2_ MCP1 concentration ($R^{2}=0.98$, with a slope of 0.613). The conversion between macrophage and *MCP*1 is, therefore, given by Equation (4):

(4) $$\log_{10}\left(M\right)=0.6301\log_{2}\left(MCP1\right)$$

where *M* is the macrophage cell count in the lung, and *MCP*1 has units of pg/mL. During parameter training, the macrophage state (Equation (3)) is fit to the log_2_ of *MCP*1 measurements. Equation (4) is then used to transform *MCP*1 predictions into estimates of macrophage counts in the lung.

### 2.3. Parameter Training

Basin Hopping (BH) [48], via SciPy [49], and Parallel Tempering Markov chain Monte Carlo (PT MCMC) [50] were employed as global optimization algorithms to train parameter values. BH rapidly identifies a single estimate of parameter values, whereas PT MCMC characterizes the parameter space over an extended number of samples. The objective function used (Equation (5)) is the weighted sum of squared error, and is referred to hereafter as energy, following Metropolis et al. [51]).

(5) $$Energy=\sum_{x=1}^{X}\sum_{t=0}^{T}\frac{{\left(M_{x,t}-O_{x,t}\right)}^{2}}{2O_{x,t}}\;$$

*M_x,t_* and *O_x,t_* are the model output and the average of triplicate observed data points, respectively, for each state, *x,* and time point, *t*, across all states, *X*, and time points, *T*. Each time point was divided by the corresponding data point, *O_x,t_*, to normalize energy values. All MCMC simulations ran across six chains of temperature (0.99, 0.9, 0.8, 0.4, 0.2, and 0.05) to ensure adequate exploration of parameter space. Parameters were unbounded, and priors were defined as uniform between zero and +∞. 

### 2.4. Model and Scenario Prioritization

Though an energy function conveys the quality of the fit achieved by parameterization for a given model, it is incapable of comparing models with varying numbers of parameters (differing degrees of freedom). The Akaike Information Criterion (*AIC*) was used to compare models with different numbers of parameters, and determine the superior model based on a tradeoff between the model’s fit to training data (energy) and the number of free parameters used to achieve the fit. The optimal model is the model that reports the lowest *AIC* value. As *AIC* is relative, a difference greater than 2 was considered significant when comparing two outcomes. *AIC* is defined [52] as:

(6) $$AIC=-2*\ln(MLE)+2*P_{free}$$

where *MLE* is the maximum likelihood estimate, and *P_free_* is the number of parameters being fit. The number of free parameters in a model depends on the scenario being considered, which is described in the Section 3. The maximum likelihood, *MLE*, is defined as:

(7) MLE=exp(−Energy)

### 2.5. Sensitivity Analysis

An extended Fourier Amplitude Sensitivity Testing (eFAST) global sensitivity analysis [53,54] was performed in Python Version 3.8.10 with Sensitivity Analysis Library (SALib) Version 1.4.5 [55] to determine the output variance of each state (Equations (1)–(3)) as a function of input variance to each parameter. The output of the method is First-Order indices that represent the outcome variance of each system state that can be attributed to the perturbation in a single parameter, *p*. High First-Order indices imply that a single parameter has a significant role in controlling system outcomes, whereas low values indicate a less significant impact. To determine the overall system sensitivities, the output variance of each state (*V*, *I*, *M*), for each parameter *p*, was determined at 100 time points between 0 and 5 days. The average of these sensitivity indices over all time points is reported.

## 3. Results

### 3.1. In Silico Screenings of Candidate Innate Immune Models Find That H5N1 and H1N1 Viruses Induce Interferon Production at Different Rates In Vivo

The goal of this work is to determine the innate immune processes that are differentially regulated in animals infected with a moderate H1N1 or severe H5N1 influenza virus. These processes can be represented as differences in the values of a parameter of the mathematical model. To identify differentially regulated processes, four biologically informed mathematical models with structural differences surrounding macrophage activity were developed, and a series of parameter fittings were performed to determine which parameter(s) must take on different values to optimally fit experimental data derived from H5N1- and H1N1-infected animals.

**Figure 1:**
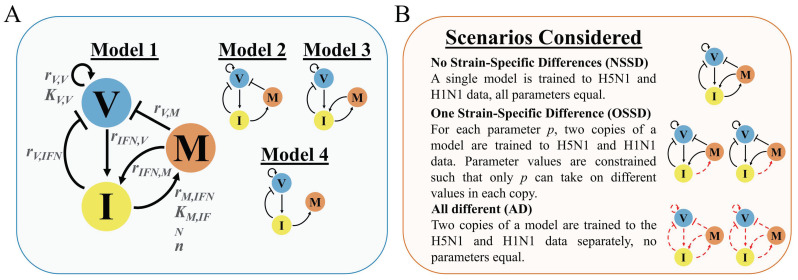
(**A**) Model schemes of the four models considered in this work. V, I, and M represent virus concentration, interferon concentration, and macrophage cell count in the lungs of infected mice. Arrows represent activating interactions; lines ending in crosses represent inhibiting interactions. The parameters involved in each interaction are indicated in Model 1 (degradation reactions not shown). Model 1 is the fully connected model of the innate immune response model. Models 2–4 are reduced versions of Model 1, wherein select interactions were removed. (**B**) Each model is analyzed for its goodness of fit to experimental data under three different scenarios. Schemes of the model emphasize the different outcomes that occur under each scenario. Black arrows indicate parameters that retain the same value when fitting the model to H5N1 and H1N1 infection data. Red, broken arrows identify parameters that take on different values when training two copies of a model to the H5N1 and H1N1 infection data.

The models are shown in Figure 1A, wherein four different regulatory structures link the concentration of virus (*V*), the level of interferon (*I*), and the number of macrophages (*M*) in the lung (see Section 2 for the rationale and mathematical equations for each model). The primary distinction between the four models involves the role of macrophages. In Model 1, macrophages can induce interferon production and suppress virus replication. However, experimental evidence suggests that macrophages may not play a major role in suppressing virus replication [47]. As such, we constructed four models of the innate immune response. In Model 2, macrophage induction of interferon production is removed. In Model 3, macrophages do not directly suppress virus replication, and in Model 4, both macrophages’ ability to induce interferon and suppress virus replication are removed.

All four models were compared under three scenarios using the *AIC* as the discrimination metric. The overall strategy of the approach is illustrated in Figure 1B. In the “No strain-specific differences” (NSSD) scenario, parameters have equal values in both infections. A single copy of each model is trained to the H5N1 and H1N1 data, resulting in one trained (parameterized) model. In the “One strain-specific difference” (OSSD) scenario, we assume that a single interaction or process may be differentially regulated in the two infections. To consider this, we train two copies of a model to the data, one copy for the H5N1 data and another for the H1N1 data, but only allow one parameter to take on different values between each copy (referred to as independent parameters, similar to the approach used in [18]). All other parameters must maintain the same value. This results in an H1N1- and an H5N1-specific parameterized version of a model, each of which has identical parameter values except for the strain-specific parameter under consideration. Lastly, we considered the “All different” (AD) scenario in which all parameters can take on different values when training a model to the H1N1 or H5N1 data, resulting in an H1N1- and an H5N1-specific parameterized version of the model in which all parameters have different values. AD provides a benchmark of the equations’ ability to capture the dynamics of each strain individually, whereas NSSD benchmarks the goodness of fit for when each infection is considered to be mechanistically identical. All four models were parameterized under each scenario using a basin hopping algorithm. *AIC* scores were used to determine which model and scenario results in the best fit to the data.

**Figure 2:**
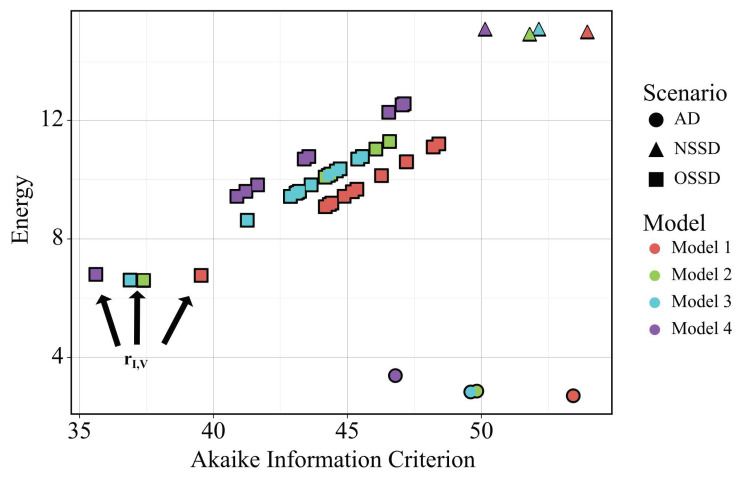
Energy versus *AIC* values for all four model structures under different parameterization scenarios (All Different (AD), One Strain-Specific Difference (OSSD), and No Strain-Specific Difference (NSSD)). The Model 4 OSSD *r_I,V_* scenario yields the global minima.

Comparing the *AIC* results after training each model to the experimental data under each scenario suggests that H5N1 and H1N1 viruses induce the production of interferon at different rates. Figure 2 shows the energy (goodness of fit) and the *AIC* for all combinations of model and scenario. Generally, all four models can attain similar goodness of fits to the immunologic data. Model 4 tends to have the lowest AIC, a result of both low energy fits and the fact that Model 4 has the fewest parameters. The lowest energy is achieved by all four models under the AD scenario, which is expected, as this scenario has the highest degree of freedom for fitting the models to the data. However, the lowest *AIC* values are not achieved under the AD scenario. The minimum *AIC* occurs in the OSSD scenario, where the parameter representative of interferon production rate, *r_I,V_*, takes on H5N1- and H1N1-specific values. All four models achieve their lowest *AIC* under this condition (noted in Figure 2), with Model 4 achieving the lowest *AIC* overall. This suggests that virus-induced interferon production is regulated in a strain-dependent manner, a proposition that is independent of the model, and therefore, macrophage activity, employed. These findings also suggest that Model 4 is the best model for regressing against the H5N1 and H1N1 immunologic data. 

### 3.2. Strain-Specific Interferon Production Is Not an Artifact of Parameter Sensitivity

**Figure 3:**
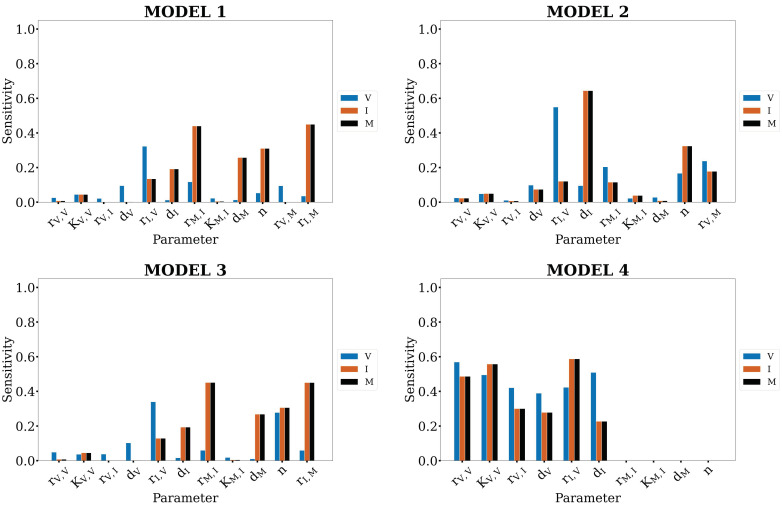
First-order indices of the eFAST sensitivity analysis for each model as described in Figure 1A. Indices are reported as the normalized change for each model state, for each parameter.

A challenge associated with this type of in silico screen is to determine if the screening methods have merely identified the most sensitive model parameter as the best parameter to take on different values and provide the best fit to the data. We next investigated the parametric sensitivity of the candidate models to determine if *r_I,V_* was the most sensitive model parameter. We conducted a sensitivity analysis of all the models to each of their constituent parameters using the eFAST algorithm [53,54]. The sensitivity of each state is reported in the form of fractional variance that can be explained by the variance of a single parameter, *p*. These indices are shown in Figure 3.

Parametric sensitivity analysis for each model shows that the most sensitive parameters differ across the candidate models. In Model 1, the concentration of interferon (*I*) and number of lung macrophages (*M*) are most sensitive to macrophage-associated parameters (*r_M,I_* and *r_I,M_*), whereas the concentration of virus (*V*) is primarily dependent on the rate of interferon induction by the virus, *r_I,V_*. This trend holds for Models 2 and 3. In Model 4, the concentration of interferon (*I*) and the macrophage count (*M*) are most sensitive to the rate of interferon induction by the virus, *r_I,V_*, whereas the concentration of virus (*V*) is most sensitive to *r_V,V_*. This establishes that the four model structures have unique control schemes, i.e., the most sensitive parameters differ between the different models. This also demonstrates that the minimum *AIC* values of *r_I,V_* OSSD models during the in silico screen were not simply the result of *r_I,V_* being the most sensitive parameter. Thus, the remainder of this work comprises further analyses using Model 4 to understand the parameter space associated with the model fitting to H5N1- and H1N1-specific data.

### 3.3. Deep Exploration of Model 4’s Parameter Space Using PT MCMC

Preliminary in silico screens and sensitivity analyses establish that Model 4 provides the best fit to the immunologic data when *r_I,V_* is allowed to take on H5N1- and H1N1-specific values. However, further exploration of the parameter space using Parallel Tempering Markov Chain Monte Carlo (PT MCMC) parameterization was needed to determine the breadth of the parameter space that supported Model 4’s best fit to H5N1 and H1N1 data. Using PT MCMC, we re-evaluated all of the scenarios described in Figure 1B for Model 4. For each MCMC optimization, 2 million iterations were run.

**Figure 4:**
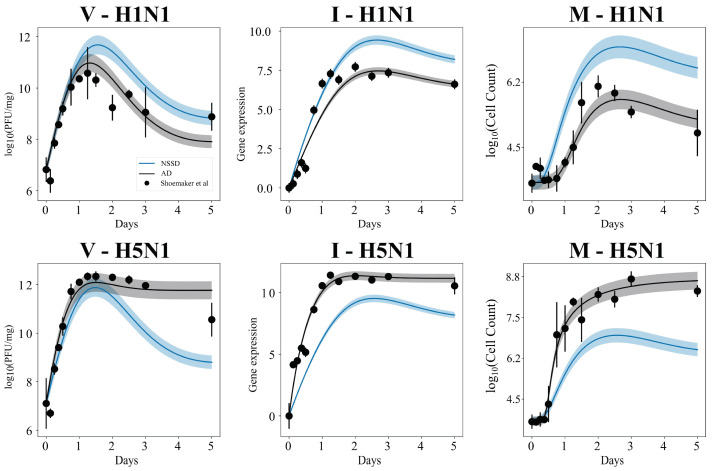
The top 1000 fits of Model 4 to the H1N1 (**top row**) and H5N1 data (**bottom row**) when using PT MCMC parameterization. The top fits under the AD scenario (all parameters allowed to independently estimate across strains) are shown in black, and NSSD results (all parameters shared between strains) are shown in blue. Intervals represent the standard deviation of the 1000 lowest energy parameter sets. Data from Shoemaker et al. [1] are shown as circles, with bars indicating the standard deviation.

Figure 4 shows the fits of Model 4 under the “all different” (AD) and “no strain-specific differences” (NSSD) scenarios plotted against the H5N1 and H1N1 in vivo mouse data. Standard deviation intervals of the top 1000 solutions (i.e., the 1000 lowest energy parameter sets that were identified) are narrow for the model’s fits under both the AD (black) and NSSD (blue) scenarios, indicating a range of possible model trajectories with similar energy. The resultant trajectory for the NSSD scenario is the average of the two strains’ datasets and, expectedly, fits neither strain. The AD scenario fits reproduce the observed dynamics for each strain very well, showing that the Model 4 equations are capable of producing known in vivo behavior, and strain-specific parameterizations can improve model energy at the cost of higher degrees of freedom.

### 3.4. MCMC-Based Parameter Exploration Again Finds That H5N1 and H1N1 Viruses Induce Interferon Production at Different Rates In Vivo

**Figure 5:**
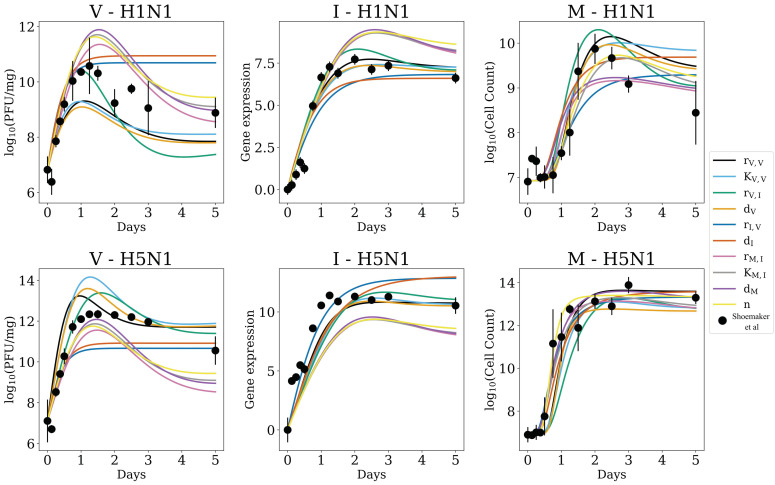
Model 4 output for the minimum energy parameter set (lines) for OSSD parameterizations and corresponding training data (markers) for H1N1 (**top row**) and H5N1 (**bottom row**). Data from Shoemaker et al. [1] are shown with the standard deviation associated with triplicate data points per time point.

We next considered Model 4’s goodness of fit to the H5N1 and H1N1 data under the OSSD scenario. The energy and *AIC* for all scenarios tested are reported in Table 1. For completeness, we show the time course trajectories of the best fit achieved for Model 4 under all OSSD scenarios in Figure 5. Energy per iteration for both AD and NSSD scenarios are shown in Supplemental Figure S3, whereas best-fit parameter values and units are provided in Appendix A. The lowest *AIC* is achieved when the rate of virus induction of interferon, *r_I,V_*, is allowed to have strain-specific values. Minimum energy values fall between 9 and 13 except in the case where the rate of interferon production, *r_I,V_,* is independently estimated, which yields a minimum energy of 6.65. Though this is closest to the minimum AD energy for Model 4 (3.33), *AIC* calculations reveal that the resulting value of 35.30 for *r_I,V_* is not only lower than the results of the other nine OSSD parameterizations of Model 4, but is lower than that of the high degree of freedom AD results. Overall, using MCMC instead of basin-hopping for data fitting did not lead to a different conclusion with regards to the optimal solution occurring when *r_I,V_* is independently estimated for H5N1 and H1N1.

**Figure 6:**
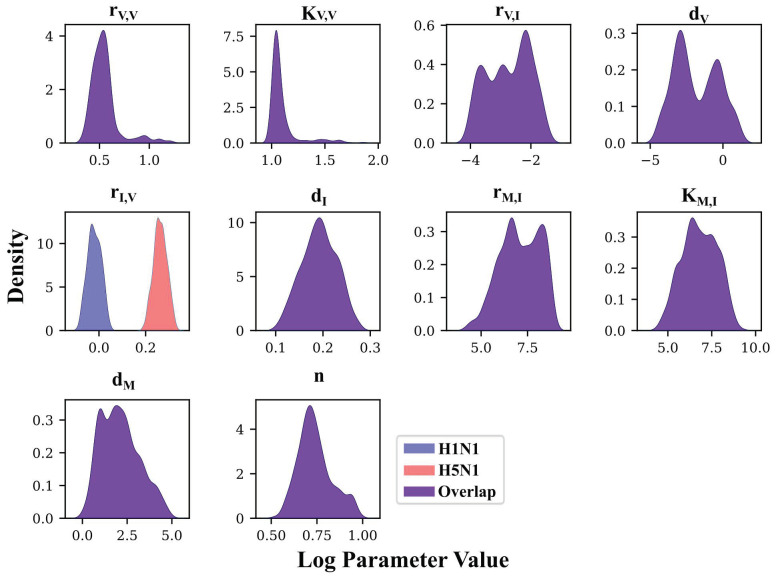
Posterior density distributions for all parameters for Model 4, with *r_I,V_* varying between strains. Only *r_I,V_* can have strain-specific values. All other parameters have the same value when fitting the model to H5N1 and H1N1 data. The x axis is given in log_10_ Parameter Value. Distributions result from the 1000 lowest energy solutions identified using PT MCMC. Narrow posterior distributions indicate that the parameter had a small range of values under which the model optimally fit the data, whereas broad distributions indicate that a range of values would yield fits of the same energy.

In total, the model fits in Figure 5 capture some trends of the in vivo data, falling short in the inability to capture V after day 1 and late infection behavior for all three states. However, the *r_I,V_* OSSD scenario shows a distinct improvement in fit over the NSSD results. When each parameter is allowed to differ between strains, histograms can inform whether the strains’ parameter distributions are unique. Focusing on the *r_I,V_* OSSD scenario histograms, a comparison of the resultant top 1000 parameter distributions across strains yields a significant difference between distribution means (Mann–Whitney test *p* < 0.001 for *r_I,V_* between H1N1 (blue) and H5N1 (red), Figure 6), indicating that the strains have unique values for this parameter. All other parameter distributions for OSSD models overlap significantly (Supplemental Figure S4), except for *d_I_*. Combined with the *AIC* results in Table 1, these results highlight that *r_I,V_* OSSD achieves the most statistically defensible fit to the datasets.

### 3.5. Independent Estimation of Virus Parameters per Strain Does Not Improve Model AIC

Because it would be computationally intractable to fit all possible combinations of parameter values, this study focused largely on observing the effect that differences in single parameters while training to two infection datasets have on model quality. However, we hypothesized that disparate immune dynamics between viral strains may be related to all virus-based rates, such as growth rate, *r_V,V_*, or death rate, *d_V_*. To test this, Model 4 was parameterized such that the viral state parameters, *r_V,V_*, *K_V,V_*, *r_V,I_*, and *d_V_* (denoted {*V}*), could take on different values when training to the H5N1 and H1N1 data, while all other parameters remained shared between strains. Six additional “Virus-Host” parameterizations were performed with the addition of one of the non-viral state parameters, {*V} +OSSD* (DoF: 15).

**Figure 7:**
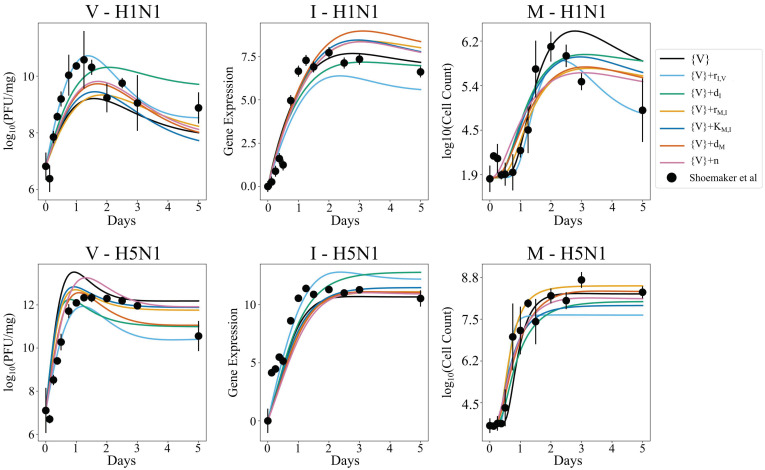
Model 4 output for minimum energy parameter set (line) for virus-related parameter independent ({*V}*) and corresponding training data (markers) for H1N1 (**top row**) and H5N1 (**bottom row**). {*V}* is representative of four viral parameters: *r_V,V_*, *K_V,V_*, *r_V,I,_*, and *d_V_*. Data from Shoemaker et al. [1] are shown with the standard deviation associated with triplicate data points per time point.

The model solutions for each parameterization are found in Figure 7. Qualitatively, the resulting fits are more indicative of the expected dynamic trends, including during late infection. Model fits follow similar trends to Figure 4 and Figure 5, with {*V}* + *r_I,V_* achieving the best fit to data. Corresponding minimum energy and *AIC* values are found in Table 2. A comparison of the top 1000 parameter distributions per strain yields significant differences between distribution means, except for *r_V,I_* in {*V} + K_M,I_* (Mann–Whitney test *p* < 0.001 for all independently estimated parameters). This indicates that virus-related kinetic parameters likely vary between strains. Minimum energies associated with the {*V}* + OSSD parameterizations are lower than that of {*V}* alone, with a minimum energy of 5.55, associated with the independent fitting of {*V}* + *r_I,V_*. Compared to the AD and NSSD scenarios, {*V}* + *r_I,V_* results in a lower *AIC* value, reiterating the role of interferon production rate in strain-specific infection dynamics. Although strain-specific viral parameters are demonstrably present in the datasets, {*V}* + *r_I,V_* has a higher *AIC* than the *r_I,V_* OSSD scenario. This attributes great importance to strain-dependent interferon production rate over simple strain-dependent viral kinetics and implies that increased degrees of freedom are detrimental to model quality. Investigations with higher degrees of freedom were not performed due to the computational time required for each MCMC fit to run 2 million samples per study.

## 4. Discussion

In this work, four distinct, three-state ODE models of the early innate immune response to influenza virus were used to investigate the mechanistic roots of differential immunoregulatory behavior observed in vivo between low- and high-pathogenic H1N1 and H5N1 strains. Several mechanisms have been hypothesized to explain differential immunoregulation between low- and high-pathology infections. Three specific mechanisms are that high-pathogenic viruses may simply replicate more quickly, high-pathogenic viruses may differently interact with antiviral signaling pathways (i.e., interferon signaling), or high-pathogenic viruses may infect and/or alter the behavior of macrophages (see introduction for further details). Prior modeling efforts implicated the infection of macrophages as a driving factor for strain-dependent pathogenicity [18]; however, the study did not consider alternative mechanisms, and further exploration was needed [46,47]. The in silico screen used here is an unbiased approach that allows several candidate mechanisms to compete, with the most likely candidate mechanism being selected based on the model’s goodness of fit to H5N1 and H1N1 training data. The infection data originate from identical lineage-, age-, and gender-matched murine subjects, minimizing inter-individual variability, and increasing the likelihood that differences observed between infections are due to strain-specific immunoregulation or virus replication.

Of the three hypotheses for why H5N1 viruses induce distinct immune responses, the primary finding from the in silico screen (Figure 2) is that the rate of interferon production by infected lung cells is likely different in H1N1- and H5N1-infected animals. The lowest *AIC* was achieved when the interferon-associated parameter, *r_I,V_*, was allowed to take on different values while training to each infection cohort—regardless of the model employed. The robustness of this finding is further supported by the wide distribution of parameter values which optimally fit the data, quantified by the MCMC analysis, and by the results of the sensitivity analysis. One concern about our in silico screening approach, and indeed in model-based analysis in general, is that the most sensitive parameters are often identified as the most important for maintaining phenotypes, as they are the easiest to use for tuning system dynamics. Across the four models considered here, the top parameters to which the model outputs are sensitive differed (Figure 3). Nonetheless, *r_I,V_* was identified as the most likely candidate across all four models. Finally, in Figure 5, MCMC analysis showed that the best fit for the scenario with strain-specific *r_I,V_* values could be achieved for a wide range of parameter values. It was found that the rate of interferon production, *r_I,V_*, is approximately 2–3 times faster in H5N1-infected lung cells. Additional analyses were performed to consider strain-specific virus replication rates combined with strain-specific immune rates (Table 2; Figure 7). Our work demonstrates that strain-dependent differences arise from host–virus interactions and immunological reactions, rather than strain-specific viral replication behavior.

It is important to note that although the in silico screen identifies strain-specific interferon production as the key mechanism for differential immunodynamics, this does not fully negate the possibility of other mechanisms. Each of the three mechanisms discussed are supported by some studies and contradicted by others. For example, with regards to macrophages, studies have shown that macrophages are susceptible to high-pathogenic viruses [56], and H5N1 viruses can replicate in human macrophages cultured from monocytes [57]. However, it has also been shown that macrophages collected from human donors can be infected by H5N1 viruses, but do not produce virus nor inflammatory cytokines [12]. With regards to strain-specific regulation of interferon, there is evidence that H5N1 viruses may upregulate interferon production early in infected cells in vitro [58]. Our in silico screen considers several possible mechanisms for why H5N1 and H1N1 immunodynamics may differ, and though we conclude that strain-specific interferon production is the most likely mechanism, we only considered two or more possible mechanisms occurring simultaneously. Given the complexity of the immune system, future efforts will focus on considering more complex candidate mechanisms.

The caveats of this study primarily relate to the available data and parameter identifiability. Insufficient macrophage count data were available, and the concentration of MCP1 was used as a surrogate measurement. Although data were available to assess the accuracy of using MCP1 as a surrogate, there remains the possibility that macrophage counts differed from our estimates. With regards to parameter identifiability, in highly connected systems, such as Model 1, it is often difficult to reasonably estimate values for all parameters. This can be improved in future work by incorporating data from knockout mice studies wherein feedback in immune signaling can be removed. The shared parameter optimization framework is highly generalizable to other cohorts of data, including age-, race-, and sex-specific studies, making it a highly valuable tool for investigating disparate kinetics between groups of interest and the drivers of observed clinical behavior [59,60]. Additionally, conclusions from cohort-specific studies may prove useful for informing and simplifying future modeling work with additional cohorts.

## Figures and Tables

**Table 1:** The minimum energy, degrees of freedom (DoF), and *AIC* values achieved by Model 4 for each scenario. The independent parameter column identifies the parameter allowed to take on different values while training two copies of the model to the H5N1 and H1N1 data.

Scenario	Independent Parameter	Energy	DoF	AIC
NSSD	None	15.04	10	50.08
AD	All	3.33	20	46.66
OSSD	$r_{V,I}$	10.83	11	43.66
	$r_{V,V}$	9.37	11	40.74
	$K_{V,V}$	9.79	11	41.59
	$d_{V}$	9.65	11	41.31
	$r_{I,V}$	6.65	11	35.30
	$d_{I}$	10.3	11	42.61
	$r_{M,I}$	12.36	11	46.73
	$k_{M,I}$	12.29	11	46.57
	n	12.28	11	46.57
	$d_{M}$	12.37	11	46.75

**Table 2:** The minimum energy, degrees of freedom (DoF), and *AIC* values for all seven viral subset ({*V}*) studies. {*V}* is representative of four viral parameters: *r_V,V_*, *K_V,V_*, *r_V,I_* and *d_V_*. Model scenarios are given in Figure 1. Independent parameter identifies the parameters allowed to take on different values when training to the H5N1 and H1N1 data.

Scenario	Independent Parameter	Energy	DoF	AIC
{*V*}	{*V*}	9.34	14	46.68
{*V*} + OSSD	$\left\{V\right\}+r_{I,V}$	5.55	15	41.11
	$\left\{V\right\}+d_{I}$	8.38	15	46.75
	$\left\{V\right\}+r_{M,I}$	8.86	15	47.72
	$\left\{V\right\}+K_{M,I}$	8.89	15	47.79
	{V}+n	8.92	15	47.85
	$\left\{V\right\}+d_{M}$	8.89	15	47.78

## Data Availability

Visualization codes for Figure 4, Figure 5 and Figure 7, which result from MCMC parameterization, can be found at https://github.com/ImmuSystems-Lab/Macrophage_Model.

## Supplementary Materials

The following supporting information can be downloaded at: https://www.mdpi.com/article/10.3390/v14050906/s1, Figure S1: Macrophage and MCP1 correlation; Figure S2: Models 2 and 3 predictions; Figure S3: AD and NSSD model 4 energy plots; Figure S4: AD, NSSD, and OSSD model 4 parameter posterior density distributions.

## Appendix A

**Table A1:** AD Minimum energy parameter values and units for each strain in Model 4.

Parameter	H1N1	H5N1	Unit
$r_{V,V}$	1.22	1.21	$days^{-1}$
$K_{V,V}$	3.65 × 10^1^	7.80 × 10^2^	$log_{10}\left(PFU/mg\right)$
$r_{V,I}$	1.20 × 10^−1^	1.07 × 10^−1^	$days^{-1}$
$d_{V}$	1.61 × 10^−1^	1.10 × 10^−5^	$days^{-1}$
$r_{I,V}$	7.70 × 10^−1^	3.06	${\left[log_{10}\left(PFU/mg\right)\;hours\right]}^{-1}$
$d_{I}$	9.59 × 10^−1^	3.22	$days^{-1}$
$r_{M,I}$	2.16 × 10^7^	9.71 × 10^3^	$\frac{Macrophage\;Cell\;Count}{days}$
$K_{M,I}$	1.90 × 10^5^	1.04 × 10^9^	*unitless*
$d_{M}$	8.80 × 10^3^	6.18 × 10^−1^	$days^{-1}$
n	5.47	9.98	*unitless*
$r_{V,M}$	N/A		${\left[Macrophage\;Cell\;Count\;days\right]}^{-1}$
$r_{I,M}$	N/A		${\left[Macrophage\;Cell\;Count\;days\right]}^{-1}$
