# Supplementary material for: Mathematical Modeling Finds Disparate Interferon Production Rates Drive Strain-Specific Immunodynamics during Deadly Influenza Infection
Source: Viruses. 2022 Apr 27;14(5):906. doi: 10.3390/v14050906 (PMC9147528; doi:10.3390/v14050906)
Supplement: Supplementary file 1 [file viruses-14-00906-s001.zip › viruses-1582244-supplementary.pdf]

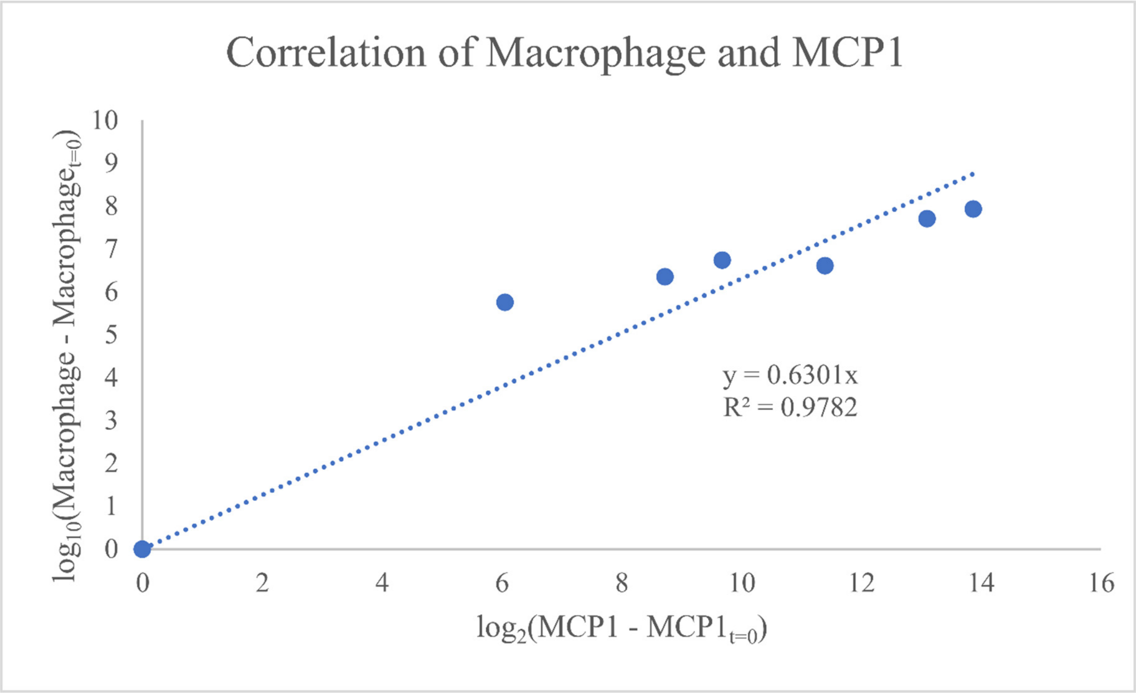

Figure S1. Macrophage and MCP1 correlation. Log_{10} of macrophage cell count in the lung is highly correlated with the log_2 of MCP1 gene expression. This regression is used to translate between MCP1 and Macrophage states.

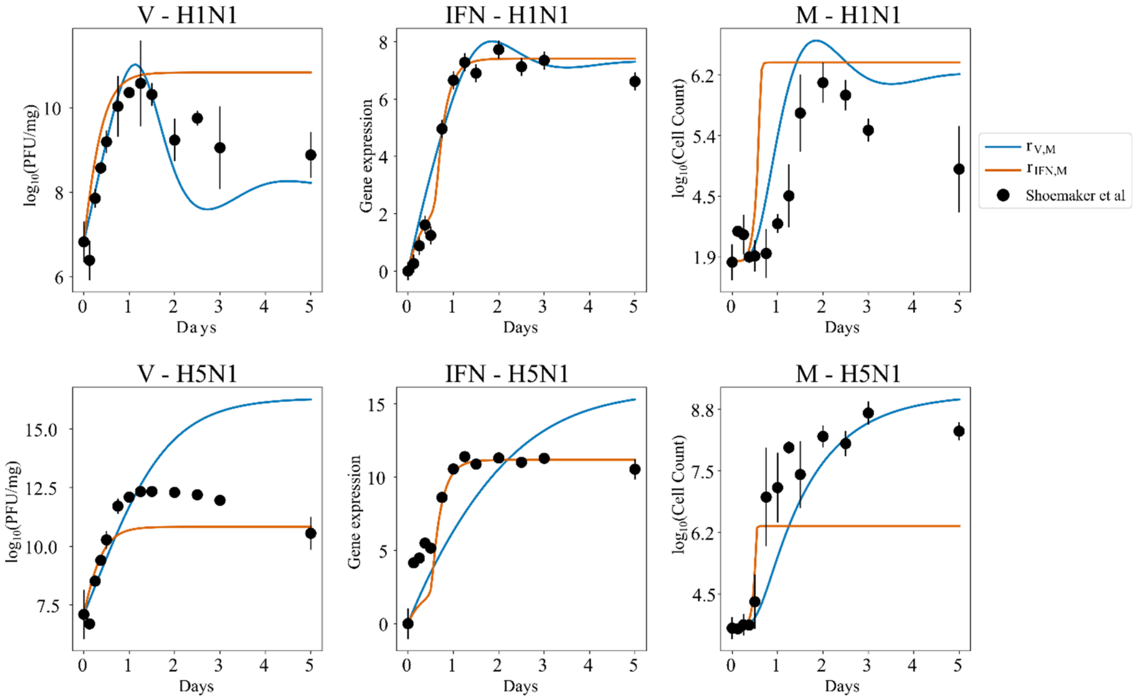

Figure S2. Models 2 and 3 predictions**.** Macrophage-dependent feedback mechanisms (Models 2 and 3 NSSD) exhibit worse fits than a model structure without these mechanisms (Model 4). Macrophage-based clearance of Virus ($r_{V,M}$, blue line) fits

H5N1 data poorly. Data from Shoemaker et al [1] are shown with the standard error associated with triplicate data points per timepoint.

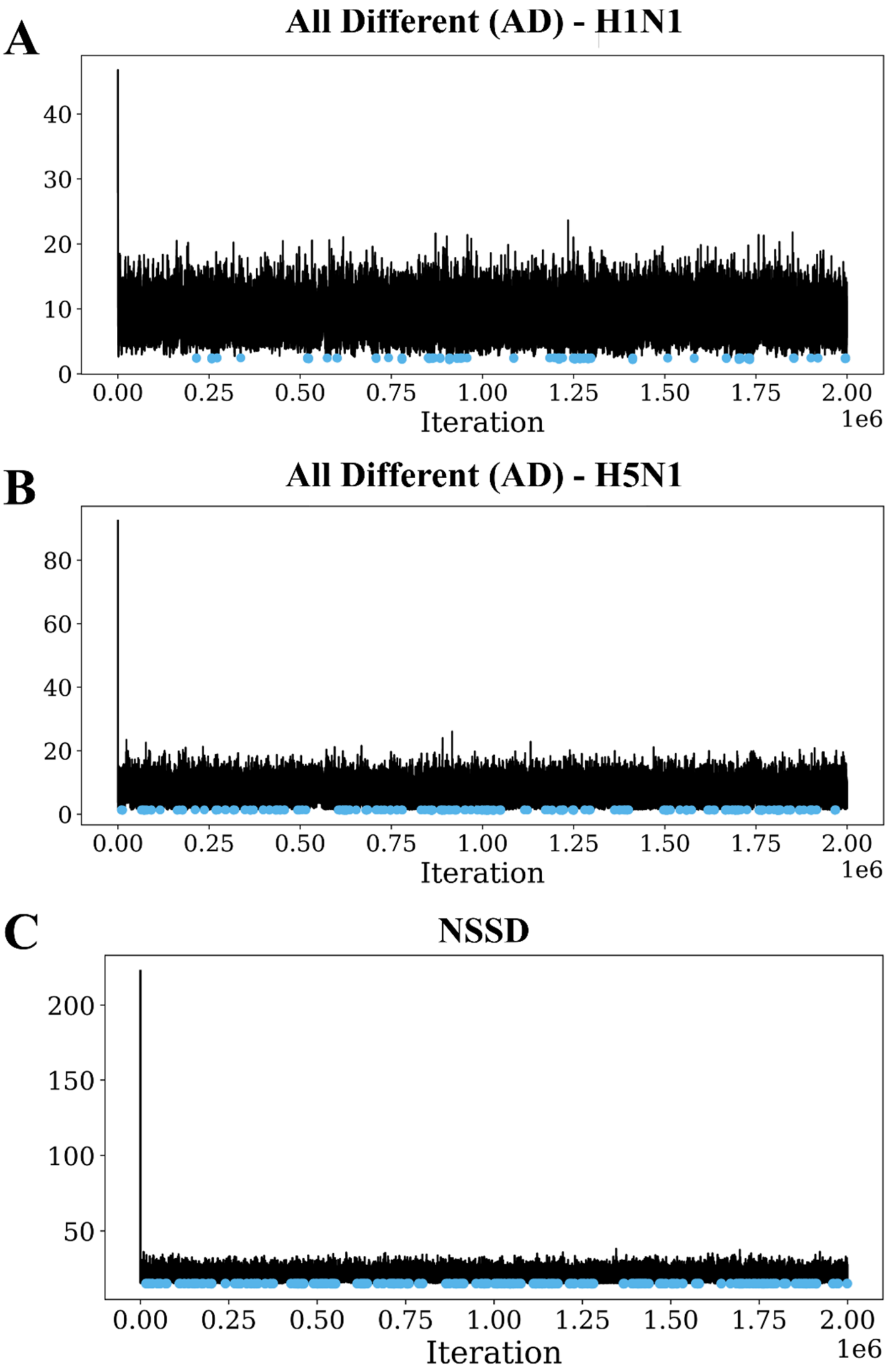

Figure S3. AD and NSSD model 4 energy plots. MCMC quickly completes burn-in and thoroughly explored parameter space for all three scenarios.

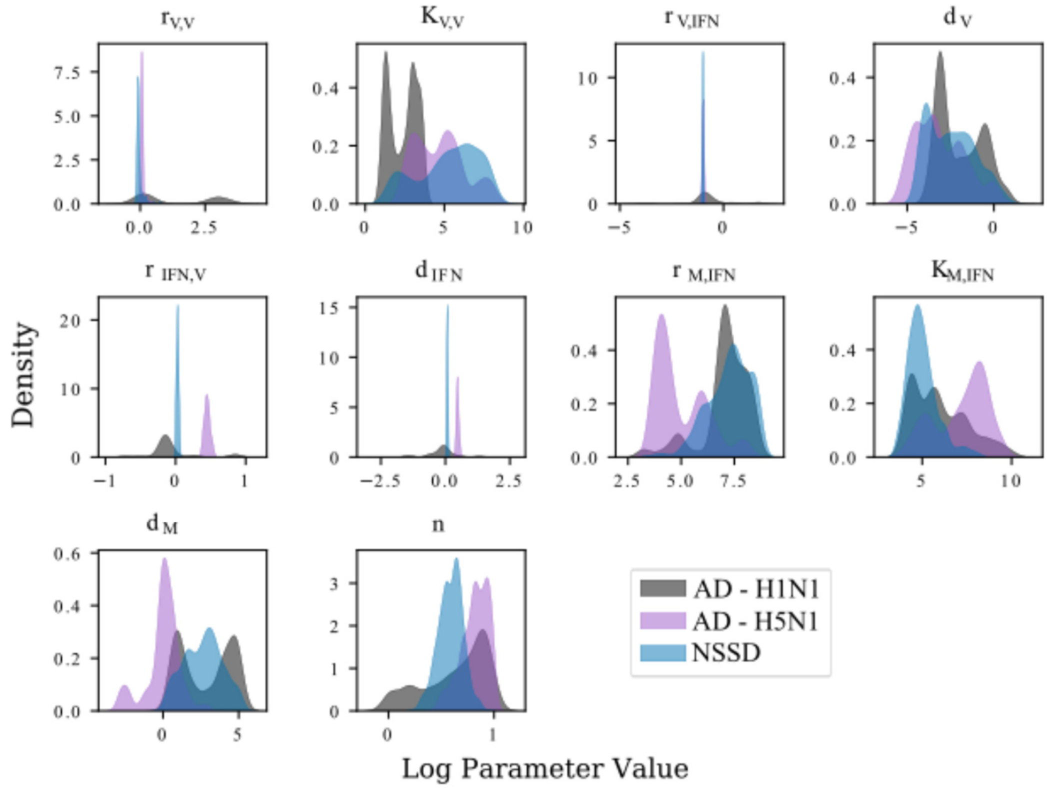
$r_{V,V}$
$K_{V,V}$
$r_{V,IFN}$
d_V
$r_{IFN,V}$
d_{IFN}
$r_{M,IFN}$
$K_{M,IFN}$
d_M
n
Density
AD - H1N1
AD - H5N1
NSSD
Log Parameter Value

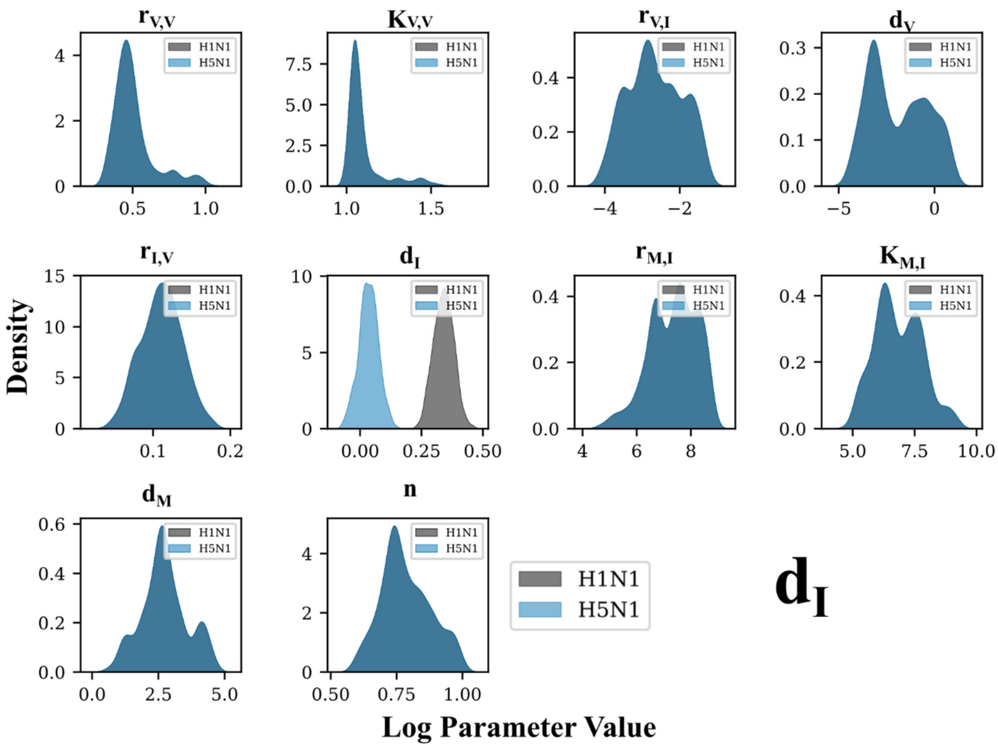
$r_{V,V}$
$K_{V,V}$
$r_{V,I}$
d_V
$r_{I,V}$
d_I
$r_{M,I}$
$K_{M,I}$
d_M
n
Density
H1N1
H5N1
Log Parameter Value
d_I

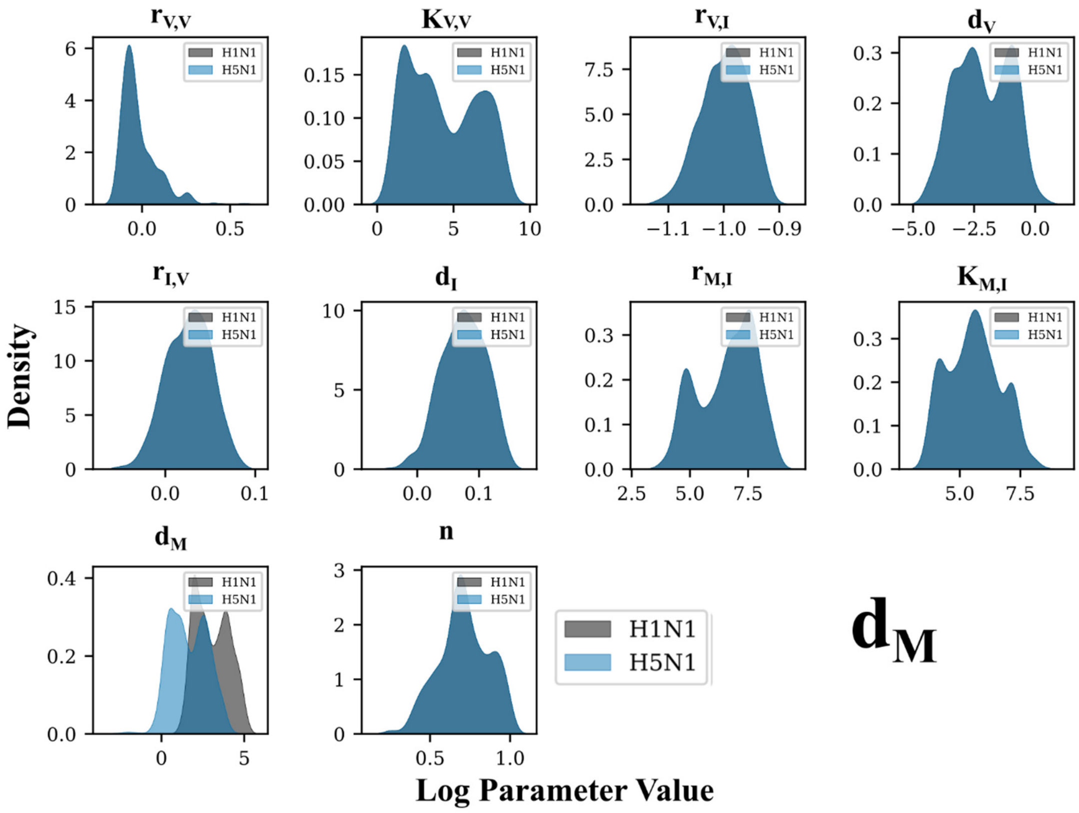

r_{V,V}
K_{V,V}
r_{V,I}
d_V
r_{I,V}
d_I
r_{M,I}
K_{M,I}
d_M
n
Density
Log Parameter Value
H1N1
H5N1
d_M

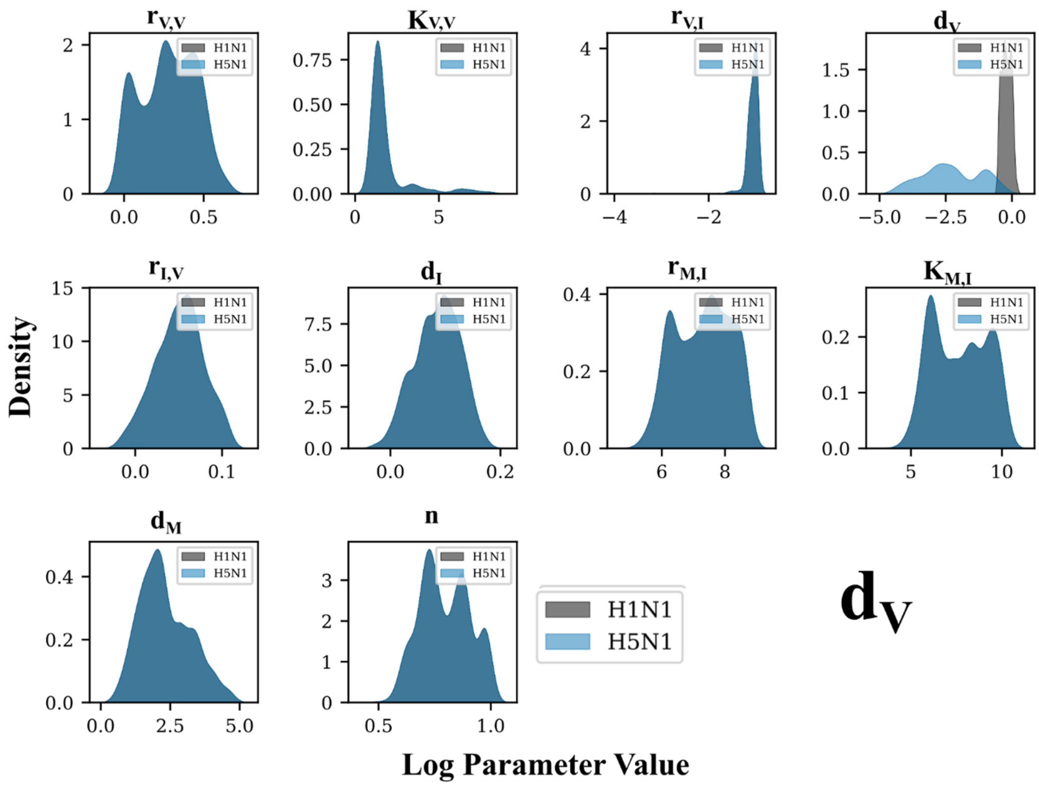

r_{V,V}
K_{V,V}
r_{V,I}
d_V
r_{I,V}
d_I
r_{M,I}
K_{M,I}
d_M
n
Density
Log Parameter Value
H1N1
H5N1
d_V

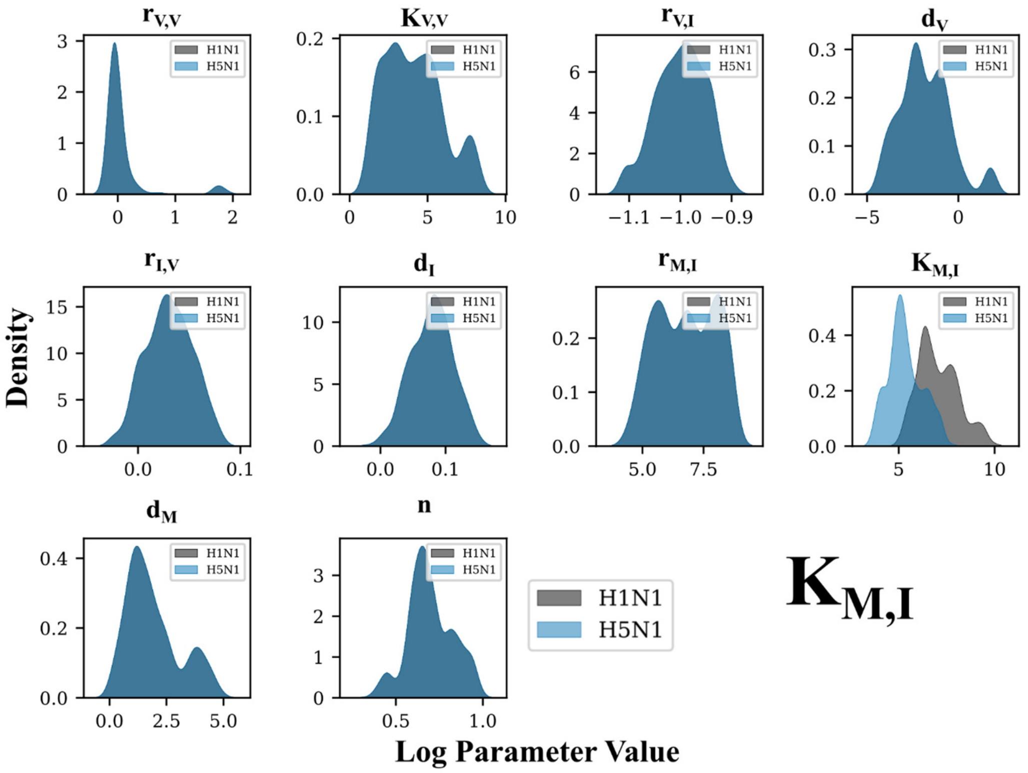

r V,V
K V,V
r V,I
d V
r I,V
d I
r M,I
K M,I
d M
n
H1N1
H5N1
Density
Log Parameter Value
K M,I

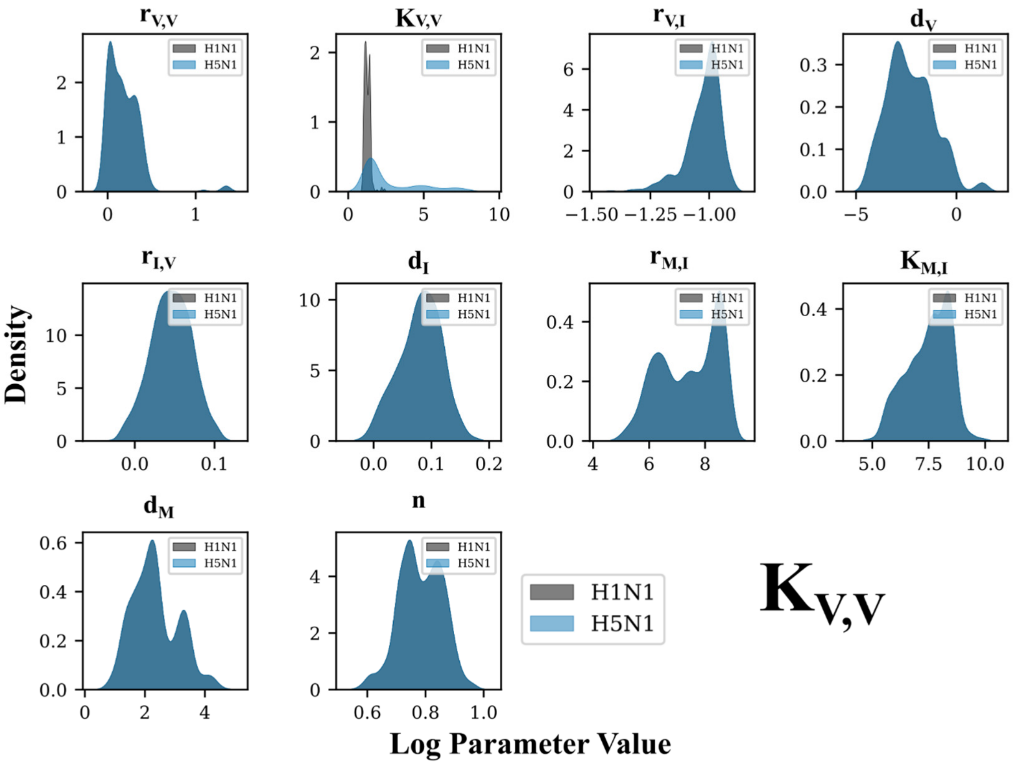

r V,V
K V,V
r V,I
d V
r I,V
d I
r M,I
K M,I
d M
n
H1N1
H5N1
Density
Log Parameter Value
K V,V

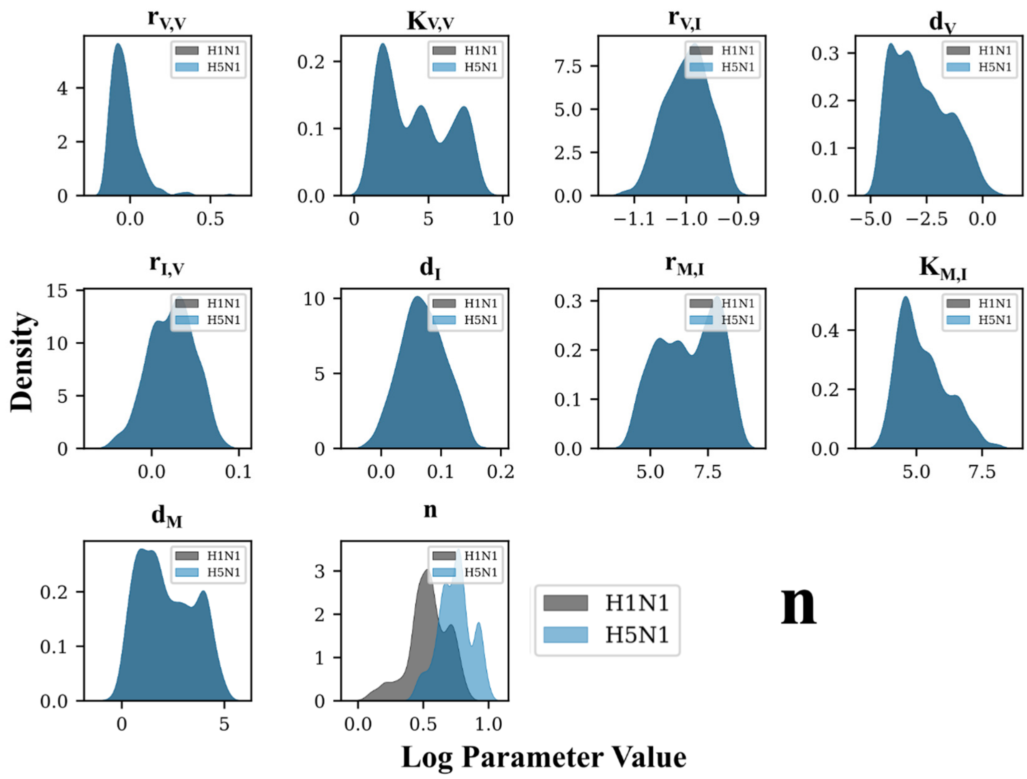

$r_{V,V}$
$K_{V,V}$
$r_{V,I}$
d_V
$r_{I,V}$
d_I
$r_{M,I}$
$K_{M,I}$
d_M
n
H1N1
H5N1
Density
Log Parameter Value
n

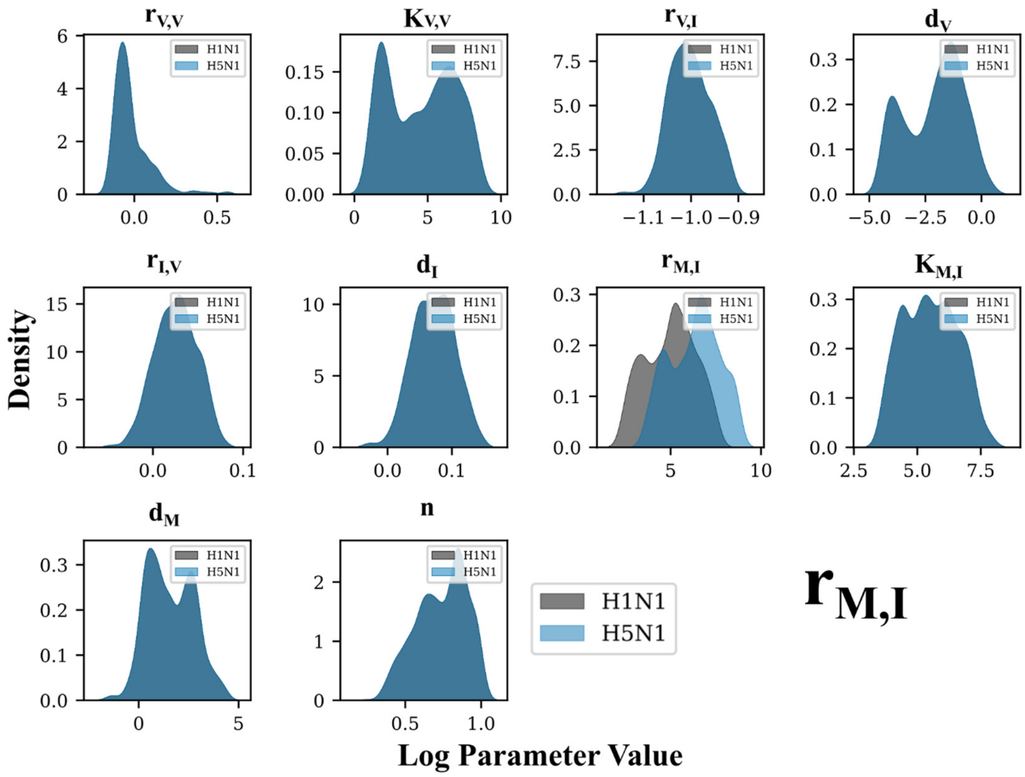

$r_{V,V}$
$K_{V,V}$
$r_{V,I}$
d_V
$r_{I,V}$
d_I
$r_{M,I}$
$K_{M,I}$
d_M
n
H1N1
H5N1
Density
Log Parameter Value
$r_{M,I}$

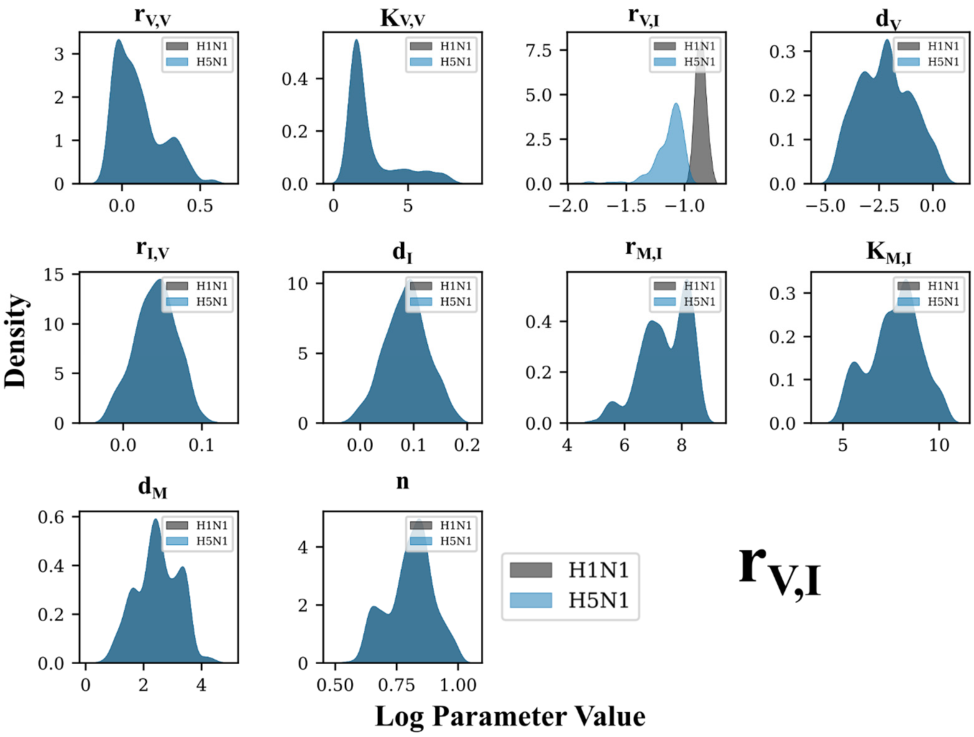

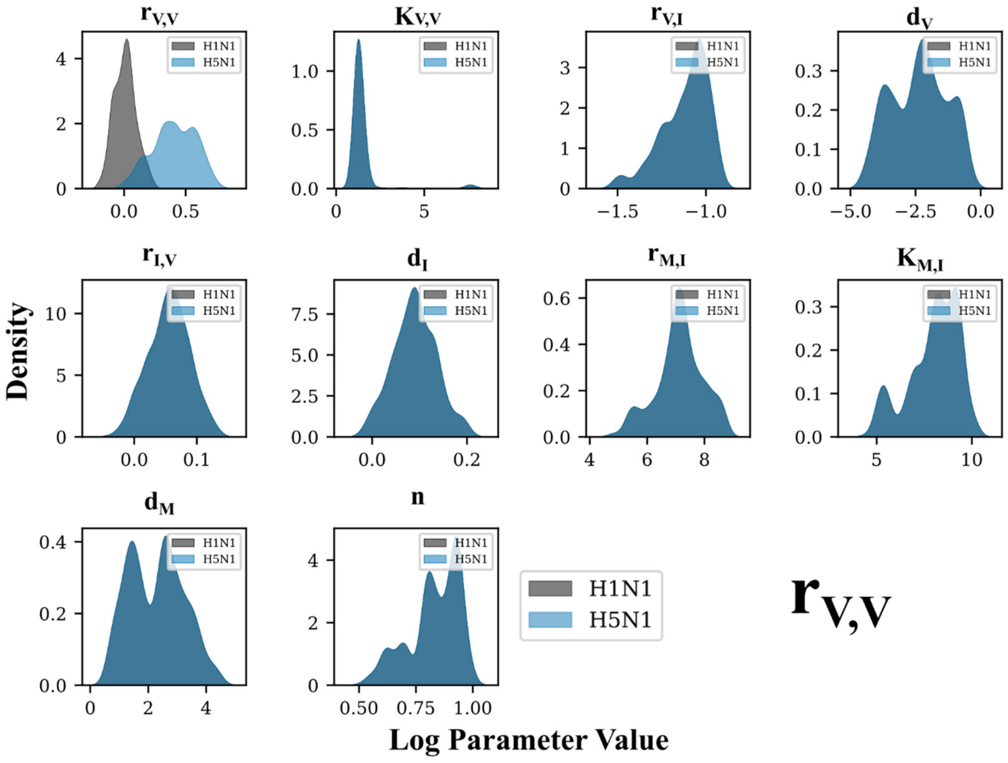

Figure S4. AD, NSSD, and OSSD model 4 parameter posterior density distributions. Overlapping distributions with an OSSD parameter (*$r_{M,I}$*, etc.) likely indicate non strain-specific mechanisms are likely, while distinct distributions (*d_I*, etc.) likely indicate strain-specific differences exist.
